# Prevalence of Hepatitis B e Antigen in Chronic HBV Carriers in North-central Nigeria

**DOI:** 10.3329/jhpn.v30i4.13289

**Published:** 2012-12

**Authors:** Joseph C. Forbi, Odunayo H Iperepolu, Timothy Zungwe, Simon M. Agwale

**Affiliations:** Clinical Virology Laboratory, Innovative Biotech, Keffi/Abuja, Nigeria

**Keywords:** HBeAg, HBsAg, Hepatitis B virus, Hepatocellular carcinoma, Nigeria

## Abstract

Hepatitis B virus (HBV) is an important clinical problem due to its worldwide distribution and potential of adverse sequelae, including hepatocellular carcinoma (HCC). We studied the prevalence of hepatitis B virus e antigen (HBeAg) among individuals determined to be HBV surface antigen-positive (HBsAg^+^) and analyzed the gender/age category associated with more active HBV infection. A total of 572 HBsAg^+^ individuals, as determined by a double antibody sandwich ELISA method, participated in the study. They were tested for HbeAg, using a lateral flow chromatographic immunoassay. One hundred and ten individuals were found to be HBeAg-positive giving an overall prevalence of 19.2%. Of these 110 individuals, 20 (18.2%) were females, and 90 (81.8%) were males. Thus, the prevalence of HBeAg appears to be higher in males than in females (p<0.05). Our data also revealed that the prevalence of HBeAg was higher in patients between the age-group of 0-10 years and 11-20 years and appeared to decrease with increase in age. Taken together, our data show that approximately 1/5 of HBV-infected individuals are HBeAg^+^, suggesting that the virus is actively replicating and infecting liver-cells thereby ensuring an HBV-transmission pool within the Nigerian population. We suggest strengthening of the childhood HBV vaccination programmes, massive intervention activities, and treatment programmes, especially among young people to reverse the possible devastating effect of HBV infection. The success of these efforts will depend on our resolution to make the elimination of HBV infection a top priority on the public-health agenda as we start the second decade of this new century.

## INTRODUCTION

In Nigeria, hepatitis B virus (HBV) infection has reached hyper-endemic levels (1,2,3,4,5). HBV is a DNA virus that replicates asymmetrically via a reverse transcription of an RNA intermediate, making it prone to mutations in the genome at an approximate rate of one nucleotide/10,000 bases/infection year (6). Hepatitis B e antigen (HBeAg) is derived from the translation product of the precore and core regions. It is selected from the infected hepatocyte because of a secretory signal sequence at the beginning of the precore region (7). Seroconversion from HBeAg to e antibody (anti-HBe) is usually accompanied with cessation of HBV replication, remission of liver disease with a decrease in serum HBV viral load and is associated with a favourable prognosis (8,9).

There are eight major genotypes of HBV, and these have been shown to have distinct geographical preference (10). HBV genotype E is almost restricted to West Africa, including Nigeria where this genotype is predominant (1,5). The expression of HBeAg may vary depending on the genotype just as clinical outcome and response to antiviral treatment in different population groups have been associated with varying viral genotypes (11). Several studies have shown that HBeAg is a biomarker of active viral proliferation in hepatocytes, infectivity, and transmission and is associated with an increased risk of hepatocellular carcinoma (12,13). In Nigeria, whenever HBeAg was present, it was associated with active liver disease, liver cancer, and death (14). Therefore, testing for the HBeAg in Nigeria can aid in identifying individuals with a high risk of developing liver cancer and in planning patient management. This can also provide information on the future burden of liver cancer associated with HBV.

Despite the widespread HBV infection and its association with liver disease in Nigeria (1,2,3),(4,5,14), limited literature about the burden of active viral infectivity is known in the Nigerian population where HBV is expanding (5). This study was undertaken to estimate the prevalence of hepatitis B e antigen-positive cases among HBV-infected individuals as determined by HBsAg seropositivity in a cross-section of people in the north-central Nigeria where HBV genotype E is predominant (1,5) and to correlate the results with gender and ages of the study participants.

## MATERIALS AND METHODS

### Study cohort

In total, 572 blood samples that were initially tested at the Innovative Biotech (IBL) clinic and known to be positive for HBsAg were randomly collected for this study. Five millilitres of venous blood was collected in a sterile container from each patient, using disposable syringe. The serum was separated and preserved at −24 °C in sterile plastic containers. The samples were collected between March 2006 and December 2009. Each patient, who attended the IBL clinic, provided informed consent to collect blood specimens and perform various serologic and biochemical assays. The use of samples for this study was approved by the IBL research committee.

### HBsAg testing

The Shantest^TM^-HBsAg ELISA (SHANTHA BIOTECH LIMITED-India) was used for the detection of HBsAg in plasma. Briefly, the Shantest^TM^-HBsAg ELISA (analytical sensitivity: 0.2 ng/mL; assay sensitivity: 99.9%; specificity: 100%) is based on the double antibody sandwich method and detects HBsAg in plasma, which is a marker of active HBV infection. The test was conducted following manufacturer's instructions and the microplates read at a wavelength of 450 nm, using the ELISA reader (BIO-RAD 2100, version 6.1, USA). The presence or absence of HBsAg was determined by relating the absorbance of the unknown sample to the cutoff value. The cutoff value is the mean of the optical density (OD) of the negative control plus the factor 0.025. Specimens with OD values greater than or equal to the cutoff value established with the negative control were considered positive while those with ODs lower than the cutoff value were recorded as negative (15).

### HBeAg test

The OnSite HBeAg Rapid Test (CTK Biotech, Inc San Diego, CA) was used for the qualitative detection of HBeAg in serum/plasma. The OnSite HBeAg Rapid Test is a lateral flow chromatographic immunoassay. The test cassette consists of: (i) a burgundy-coloured conjugate pad containing mouse anti-HBeAg antibody conjugated with colloid gold (HBeAb conjugates) and (ii) a nitrocellulose membrane strip containing a test band (T band) and a control band (C band). The T band is pre-coated with non-conjugated anti-HBeAg antibody, and the C band is pre-coated with goat antimouse IgG antibody. When an adequate volume of test specimen is dispensed into the sample well of the cassette, the specimen migrates by capillary action across the test cassette. HBeAg, if present in the specimen, will bind to the HBeAb conjugates. The immunocomplex is then captured on the membrane by the pre-coated non-conjugated anti-HBeAg antibody, forming a burgundy-coloured T band, indicating an HBeAg-positive test result. Absence of the T band suggests a negative result. The test contains an internal control (C band) which should exhibit a burgundy-coloured band of the immunocomplex of goat antimouse IgG/HBeAb-gold conjugates regardless of the presence of coloured T band. Otherwise, the test result is invalid, and the specimen must be retested with another device. The instructions of the manufacturers of the test-kits were strictly followed.

Data analysis was performed using PASW statistics 18. The chi-square test was used for evaluating the significance in the distribution by gender and age among those who were HBeAg-seropositive; p values of <0.05 were considered to be the level of significance.

## RESULTS

**Figure 1.**
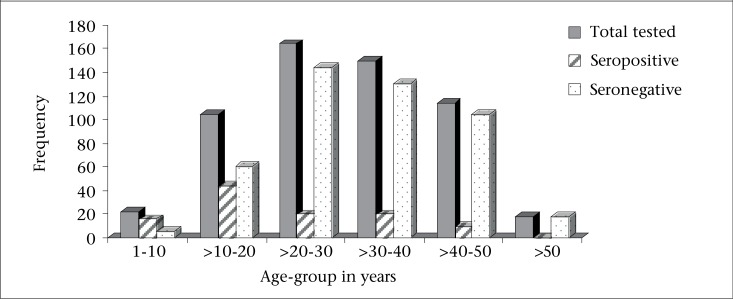
Distribution of HBeAg seropositivity and seronegativity according to age-group

**Figure 2.**
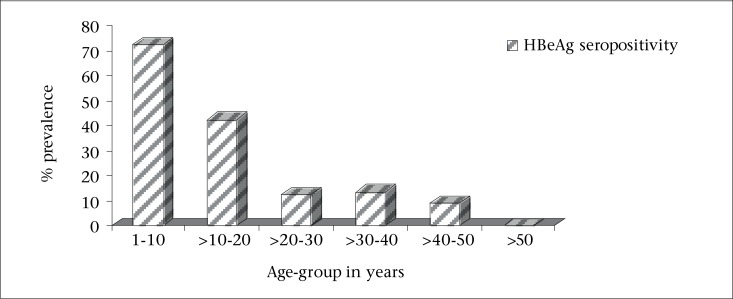
Prevalence (%) of HBeAg seropositivity according to age-group

In total, 572 HBsAg-positive individuals participated in this cross-sectional study. Their ages ranged from 3 to 64 years (mean=32±1 years). Males (n=388) dominated females (n=184) with a ratio of approximately 2:1. Of the 572 participants tested for HBV e antigen status, 110 were found to be positive (19.2%), and 462 were negative (80.8%). Among the 110 participants in the HBeAg-positive category, 20 were females, and 90 were males while 164 and 298 were females and males respectively in the negative category (n=462). Most positive cases were found to be males (81.8%) than females (18.2%) in the ratio of approximately 5:1 (p<0.05). The rate of seropositivity was categorized according to age-groups least and most affected. The number of individuals in each age category was as follows: 0-10 year(s)=22; >10-20 years=104; >20-30 years=164; >30-40 years=150; >40-50 years=114; >50 years=18 (Figure 1); the percent seropositivity by age-group was 72.7%, 42.3%, 12.2%, 13.3%, 8.8%, and 0% respectively. This could be better visualized as a proportion of the total number tested and as the prevalence recorded in percentages (Figure 1). The prevalence of HBeAg seropositivity was the highest in individuals between the age-group of 0-10 year(s) and >10-20 years (Figure 1 and 2). Individuals between the age-group of >20-30 years, >30-40 years, and >40-50 years showed higher seronegativity (Figure 1 and 2). Younger individuals tended to be HBeAg-positive than their older counterparts.

**Figure 3.**
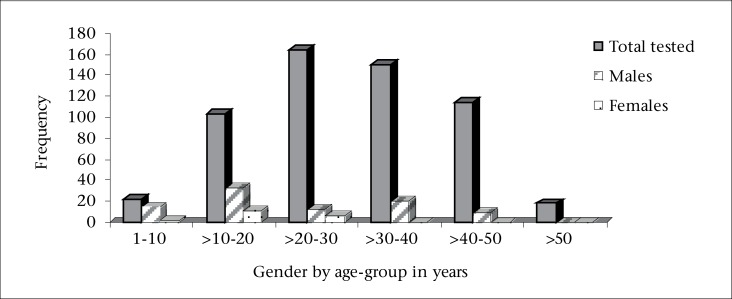
Distribution of HBeAg seropositivity according to gender

Further sub-categorization of HBeAg seropositivity by gender and age-groups were as follows: 1-10 year(s) (14 males and 2 females), >10-20 years (33 males and 11 females), >20-30 years (13 males and 7 females), >30-40 years (20 males and 0 female), >40-50 years (10 males and 0 female), >50 years (0 males and 0 females) (Figure 3). In all age-groups, the male counterparts dominated in terms of HBeAg seropositivity, suggesting a likely poorer outcome of HBV infection among males. This study was clinic-based, and since individuals with symptoms of HBV infection would present most frequently in clinics, we are unable to state categorically if the differences in gender preponderance were real or due to differences in healthcare-seeking behaviours among the north-central Nigerians. Patients in clinical settings are more likely to have medical conditions (symptoms of hepatitis), including the presence of hepatitis B virus e antigen.

## DISCUSSION

HBV is classified into eight genotypes (designated by letters A-H) on the basis of divergence of 8% or more in the nucleotide sequence, and they have distinct geographical distributions (10). Genotype E is almost restricted to West Africa, including Nigeria where this genotype is predominant (1,5). The expression of HBeAg may vary depending on the genotype just as clinical outcome and response to antiviral treatment in different population groups have been associated with varying genotypes (11). The problem of HBV infection in Nigeria is well-recognized, especially among virologists and hepatologists but efforts to control the virus have not been satisfactory as a significant impact on disease incidence or prevalence has not been observed. The presence of HBeAg in the serum of patients with hepatitis B virus is a reflection of active viral replication in hepatocytes and is considered a surrogate marker for the presence of the DNA of hepatitis B virus (13). Testing for the HBeAg can also identify individuals with a high risk of developing liver cancer (12,13). In this study, 19.2% HBeAg prevalence was recorded among HBsAg-positive individuals in north-central Nigeria. This reflects a pool of individuals who are highly infectious and serve in sustaining viral transmission and evolution in the Nigerian population, suggesting that the future burden of liver cancer associated with HBV is likely to be high. A previous study among pregnant women in Makurdi in north-central Nigeria found that 30.3% were HBV carriers testing positive for HBeAg (16). The above-mentioned study and the present study probably reflect a high proportion of HBV infectivity and transmissibility rates in this savanna zone of Nigeria.

Contrary to this high prevalence of HBeAg among inhabitants of the savanna zone of Nigeria, comparatively lower prevalence of HBeAg has been recorded among inhabitants of the most southerly forest zone of the country. In a recent study in Enugu (17), low prevalence at 8.6% was found among asymptomatic adults. In Benin city, the overall HBe-antigenaemia prevalence among adults was 7.3% (18). Among blood donors in Ibadan, Otegbayo *et al.* (14) found that 4 of 37 HBsAg (10.8%) were HBeAg-positive. Patients with a clinical, ultrasound and/or histological evidence of liver cirrhosis or HCC in Lagos (19) and prisoners in south-eastern Nigeria (20) showed that HBeAg was present respectively in 11.9% and 16.4% HBsAg-positive individuals. From these studies, it appears that there is a marked geographical difference in the prevalence of HBeAg among HBsAg-positive patients in Nigeria, with the savanna zone of the broadly northern Nigeria showing a higher prevalence when compared with the southern forest zone. This, however, seems not to be the case. Ojo *et al.* (21) in Ile-Ife and Ola *et al.* (22) in Ibadan, in a dramatic twist, reported 48.4% and 19% HBeAg seropositivity among HBsAg-positive patients—all in the forest zone of Nigeria. Taken collectively and following documented evidence in Nigeria, it is clear that high infectivity of the virus is widespread among Nigerians with HBV infection (22) and whenever HBeAg was present in Nigeria, it was associated with active liver disease (18). It is, therefore, important that healthcare facilities in Nigeria should attempt to implement routine HbeAg testing of blood of all HBV-positive patients to determine the status of infection and to adequately plan for proper patient management.

In this study, we report that 81.8% of all HBeAg-positive individuals were males. There seem to be preponderance for males to have active HBV infections. This correlates with studies (from Enugu and Benin city, Nigeria), which found that the number of HBeAg-positive males were more than double the number of females (16,17). However, no inter-gender difference in HBeAg seroprevalence was observed in a study in south-eastern Nigeria (20). This contradiction could be due to the fact that our study was clinic-based while the Enugu study (20) was community-based. Patients in clinical settings are more likely to have medical conditions (e.g. symptoms of hepatitis, presence of HBeAg) than persons living in the communities. So, it remains to be determined whether the gender differences observed in this study is the true reflection of the gender distribution or is associated with the study population or the healthcare-seeking patterns of Nigerians in this region.

In this study, younger carriers (Figure 1) tended to be more frequently HBeAg-positive than older patients. This is supported by studies from Japan, which showed that the prevalence of HBeAg positivity decreased with age (23).

### Limitations

A possible limitation of this study is that a proportion of infected individuals are HBeAg-negative because they are infected with HBV variants that are unable to produce high amounts of the excreted protein that bears the HBe epitope due to mutations in the precore or core promoter regions of the HBV genome (24). Sequencing for the detection of these mutations in the precore or basal core promoter regions was not done. These mutations prevent or diminish HBeAg formation by an otherwise normally replicating hepatitis B virus. Considering this, the actual number of active HBV infection might have been underestimated in this study. Also, liver histology in HBeAg-positive HBsAg carrier children generally reveals very mild inflammation and fibrosis (25). Although these children are immune tolerant to HBV, the virus remains highly replicative and the children are highly infectious; thus, they are an important source of infection in the family and the community. The influence of this in a predominantly HBV genotype E region is not immediately clear. It appears that HBeAg is prevalent in areas where HBV genotype E is dominant. Should this be the case, the economic and medical benefits of widescale testing for HBeAg in the HBV genotype E crescent (26) would need to be evaluated and proactive measures taken in anticipation of a large-scale preventive and treatment needs.

### Conclusions

We have shown that approximately one-fifth of HbsAg-positive individuals are also positive for HbeAg, which is a marker of active viral replication and transmission. This may be taken as an indication of active HBV in the Nigerian population. This is unacceptable for a disease, against which an effective vaccine has been available since 1982. We suggest strengthening of the childhood vaccination programme, massive intervention activities, and treatment programmes to reverse the possible devastating effect of HBV infection. The success of these efforts will depend on our resolution to make the elimination of HBV infection a top priority on the public-health agenda in sub-Saharan African nations as we start the second decade of this new century.

## ACKNOWLEDGEMENTS

The authors wish to thank Dr. Entonu Peter (Medical Officer) and Victoria kemi Olaleye (Senior Nurse Psychologist) for advice and assistance in the patient recruitment. The findings, interpretations and conclusions expressed in this paper are entirely those of the authors and do not necessarily represent the views of the institutions where they work for or are affiliated with.
